# The Disproportionate Burden: Health and Economic Outcomes of COVID-19 for Native American Communities

**DOI:** 10.21203/rs.3.rs-9452333/v1

**Published:** 2026-05-27

**Authors:** Thomas Wilk, Emilia Simeonova, Randall Akee, Stephanie Carroll, Ninez A. Ponce

**Affiliations:** National Bureau of Economic Research; Johns Hopkins University; Harvard University; University of Arizona; University of California Los Angeles

**Keywords:** Long-COVID, American Indian, Rural Health, Socio-Economic Determinants

## Abstract

**Background:**

American Indian and Alaska Native (AIAN) populations experienced disproportionate health and economic impacts during the COVID-19 pandemic. While prior research has documented elevated infection and mortality rates among Indigenous communities, less is known about the persistence of COVID-19 symptoms and their potential economic consequences. This study examines differences in COVID-19 infection, long-COVID symptoms, and pandemic-related economic hardship among AIAN populations in California.

**Methods:**

We analyzed adult responses from the California Health Interview Survey (CHIS) from 2021–2023, including the public-use files and a restricted oversample of AIAN communities. Multivariate generalized linear models were used to estimate associations between demographic, socioeconomic, and health characteristics and seven COVID-19 related outcomes, including infection, long-COVID symptoms, vaccination status, testing behavior, food insecurity, job loss, and reductions in work hours to produce survey weighted population-representative estimates.

**Results:**

AIAN respondents reported higher rates of COVID-19 infection and long-COVID symptoms than non-AIAN respondents. Estimates indicate 47% of AIAN respondents reported testing positive for COVID-19 and 40% reported long-COVID symptoms, compared with 30% among non-AIAN respondents. Obesity and poverty were positively associated with infection and long-COVID symptoms. Long-COVID symptoms were positively associated with food insecurity and job loss, while vaccination was associated with lower probabilities of job loss and reductions in hours or income.

**Conclusions:**

Persistent COVID-19 symptoms may contribute to ongoing economic vulnerability in AIAN communities. Structural factors including poverty, chronic health conditions, and limited access to healthcare amplify the long-term health and economic consequences of the pandemic.

## Introduction

The COVID-19 pandemic exposed and amplified longstanding health inequities across the Americas. In the United States, American Indian and Alaska Native (AIAN) communities experienced some of the most severe health consequences during the early years of the pandemic. National surveillance data indicate that AIAN populations had substantially higher rates of infection, hospitalization, and mortality compared with the general population. AIAN communities experienced an infection rate of 20% from 2020–2022, with an age-adjusted mortality rate of 62.5 per 100,000 by the end of 2020.^1^ At this time, AIAN communities had approximately 1.6 times the number of confirmed cases, 2.4 times the number of hospitalizations, and twice the mortality rates associated with COVID-19 relative to the U.S. population overall.^2^ These disparities reflect persistent structural inequities in health care access, economic resources, and public health infrastructure that have historically affected Indigenous communities across North America.^3^ The immediate impacts of COVID-19 in the general population are well researched, however there has been limited focus on AIAN communities and less is known about long-term health and economic impacts of COVID-19 infection.^4^ This lack of data creates a void in policy, limiting Tribal Nations and community leaders’ ability to make informed public health decisions and policies.

A growing literature documents the disproportionate burden of COVID-19 among AIAN populations. Early research highlighted elevated infection rates on Tribal reservations and identified several structural risk factors, including overcrowded housing, limited access to clean water, and constrained health care capacity in rural areas.^5,6^ Other studies have documented the role of social determinants such as poverty, employment in high-contact occupations, and geographic isolation in shaping pandemic vulnerability among Indigenous populations.^7^ These structural factors intersect with long-standing disparities in chronic health conditions, including obesity, diabetes, and cardiovascular disease, which increase the susceptibility to severe COVID-19 health outcomes and increase the risk of health-related shocks to economic wellbeing.

Despite this research, important gaps remain in understanding the broader health and socioeconomic consequences of the pandemic among AIAN communities. Much of the existing evidence focuses on infection rates, hospitalization, and mortality during the early stages of the pandemic. Relatively little is known about post-acute health consequences, including persistent symptoms commonly referred to as long-COVID, or about how pandemic-related health shocks may translate into economic instability and household financial hardship. Long-COVID is increasingly recognized as a major public health challenge that can reduce labor supply, impair productivity, and increase health care utilization.^8,9^ These consequences may be particularly severe for historically marginalized populations that already face barriers to health care access and stable employment.^7^

Another challenge in understanding pandemic outcomes among AIAN populations is the persistent underrepresentation of Indigenous peoples in population health datasets. Many national surveys lack sufficient sample sizes to produce reliable estimates for AIAN communities, leading to what some scholars have described as the “statistical invisibility” of Indigenous populations in public health research.^10^ This limitation has contributed to gaps in evidence that can inform policy responses tailored to Indigenous communities. To address these gaps, this study uses data from the California Health Interview Survey (CHIS), the largest state-representative health survey in the United States, combined with a dedicated oversample designed to increase representation of AIAN populations in rural communities. California is home to the largest population of AIAN individuals in the United States and contains both urban and rural AIAN communities, providing a unique setting for examining pandemic experiences across diverse settings.

Using these data, we investigate several dimensions of the COVID-19 experience among AIAN populations. First, we assess differences in COVID-19 testing, infection, vaccination uptake, and the prevalence of long-COVID symptoms. These outcomes capture different stages of pandemic susceptibility and impact, ranging from infection risk to preventive health behaviors and persistent health consequences. Second, we examine whether pandemic-related health outcomes are associated with indicators of economic hardship, including food insecurity, job loss, and reductions in work hours or income. These outcomes reflect potential downstream socioeconomic consequences of pandemic-related health shocks. Previous researchers have noted the link between income shocks, poverty, and healthcare utilization, access, and long-run outcomes.

## Literature Review

The unequal impact of COVID-19 across racial and ethnic groups in the United States has been widely documented. Early studies found that racial and ethnic minority populations experienced higher rates of infection, hospitalization, and mortality during the first phases of the pandemic.^1,2^ Goldman et al.^14^ reports a seven-year decrease in AIAN individual life expectancy from 2019 to 2021 from 72 years to 65, with minimal recovery in 2022. Mueller et al.^15^ also find rural communities in the West were severely impacted by the pandemic, affecting employment, life satisfaction, mental health, and economic outlook. These disparities persist in the take-up of COVID-19 vaccinations when they became available.^16^ Lower vaccination rates are attributed to lower education, sociopolitical factors, employment type, and limited access to healthcare providers. Rodriguez-Lonebear et al.^6^ report that rural communities with lower proportions of homes with complete indoor plumbing were more likely to experience higher COVID-19 infection rates.

Several mechanisms have been proposed to explain the disproportionate burden of COVID-19 among AIAN populations. Structural determinants of health, including poverty, housing conditions, employment patterns, and reduced access to health care play a central role in shaping vulnerability to infectious disease outbreaks. Rural communities and Tribal populations often face geographic barriers to care, including limited access to hospitals, primary care providers, and specialty services.^7^ Health care access is further constrained by underfunding of the Indian Health Service and other Tribal health systems, which historically operate with fewer resources than other public health institutions.^17^

Pre-existing health conditions further increase vulnerability to severe COVID-19 outcomes. AIAN populations experience higher prevalence of chronic conditions such as obesity, diabetes, and cardiovascular disease, which are associated with increased risk of severe illness following infection.^3^ These disparities reflect broader social and structural determinants of health that have shaped Indigenous health outcomes over generations.^18,19^ They persist even when access to care is equalized. Medicaid beneficiary data from New York shows that AIAN individuals were more likely to be hospitalized and die from COVID-19 even though their vaccination rates were similar to white Medicaid beneficiaries.^20^

Vaccine hesitancy among minority populations is often influenced by historical experiences of medical mistreatment, structural racism, and mistrust of health institutions.^21^ Some AIAN communities also achieved high vaccination uptake through Tribal leadership, culturally grounded communication, and community-based delivery strategies, underscoring the importance of Indigenous governance and local trust in public health response.^22^ These factors highlight the importance of trust, access, and culturally appropriate outreach in shaping pandemic responses within Indigenous communities.

Beyond acute infection risks, the long-term health consequences of COVID-19 have emerged as a major economics cost factor. Recent estimates indicate that long-COVID economic effects may have cost the U.S. the equivalent of 17 percent of pre-COVID (2019) GDP.^23^ Long-COVID refers to persistent symptoms lasting weeks or months after initial infection, including fatigue, respiratory complications, and neurological symptoms. Persistent post-acute symptoms can exacerbate existing health conditions and increase long-term health care needs.^8,24^ In addition, long-COVID has been associated with reduced productivity, increased health care utilization, and additional caregiving burdens within households.^9^ Despite growing recognition of long-COVID as a public health challenge, relatively little research has examined its prevalence or socioeconomic implications among AIAN populations.

The COVID-19 pandemic generated widespread economic disruption. Job losses, reduced work hours, and increased food insecurity were common during the early phases of the pandemic, particularly among workers in sectors requiring in-person contact.^25,26^ Research on rural communities has shown that the pandemic negatively affected employment opportunities, life satisfaction, and economic outlook in many regions of the United States.^15^ Lindeboom et. al^27^ found causal impacts of adverse health shocks on male employment rates at age 40 that are about 23 percentage points lower due to unexpected disability shocks. Similarly, health shocks associated with COVID-19 infection or long-COVID may further exacerbate economic instability by limiting individuals’ ability to work or maintain stable employment.

Despite increasing attention to pandemic disparities, empirical evidence on the combined health and economic impacts of COVID-19 among AIAN populations remains limited. Oversampling Indigenous communities within large population surveys provides one strategy for addressing this limitation and improving the availability of evidence on Indigenous health outcomes. Building on this approach, this study uses data from the California Health Interview Survey (CHIS) and a dedicated oversample of AIAN communities to examine multiple dimensions of the COVID-19 experience.

### Data

#### California Health Interview Survey (CHIS)

##### Public Use Files (PUF) 2021–2023

This study uses data from the California Health Interview Survey (CHIS), the largest state-representative health survey in the United States. CHIS collects detailed information on health status, health care access, and socioeconomic characteristics of California residents through annual population-based surveys. We use data from the CHIS adult public-use files (PUF) for survey years 2021 through 2023. These surveys collectively include more than 20,000 respondents annually and are designed to produce representative estimates of the California population through the use of survey weights.^28^

The CHIS adult survey contains detailed information on demographic characteristics, socioeconomic status, health conditions, and health care access. For this study, we focus on five broad categories of variables: demographic characteristics, body weight status, poverty status, health care access, and COVID-19-related health and economic outcomes.

The COVID-19 testing questions were asked in survey years 2021–2022, while questions regarding positive COVID-19 test results and food insecurity were included in surveys from 2021 through 2023. Information on COVID-19 vaccination status and long-COVID symptoms was collected in the 2022–2023 surveys. Questions regarding employment impacts from the pandemic, including job loss and reductions in work hours or income, were included in the 2021–2022 surveys, while measures of severe food insecurity were available across all survey years.

Respondents are classified as AIAN if they self-identify as AIAN as their primary racial identity in the survey. Because the CHIS public-use files do not provide detailed information on multiracial identification, we additionally classify respondents as AIAN if they report coverage through the Indian Health Service (IHS) but do not otherwise identify as AIAN in the race variable. Since IHS eligibility is limited to members of federally recognized Tribal Nations and their descendants, this classification helps capture AIAN individuals who may not report AIAN as their primary racial category within the survey.

All analyses apply the survey weights provided by CHIS to account for the complex sampling design and to produce population-representative estimates.

##### Restricted Oversample (RO) 2021–22

Indigenous populations are often underrepresented in national and state population health surveys due to relatively small population sizes and geographic dispersion. To improve representation of Indigenous populations, CHIS conducted a dedicated oversample of AIAN communities across the 2021–2022 survey years. This restricted oversample (RO) targeted AIAN communities and nearby rural populations located near Tribal lands and areas with higher Indigenous population density.

The oversample substantially increases the number of AIAN respondents available for analysis and provides improved representation of Indigenous communities, particularly those located in rural areas. The RO includes a larger proportion of rural respondents relative to the standard CHIS public-use files, with approximately 23% of respondents residing in rural areas compared with 9–13% in the PUF survey data.

For descriptive comparisons, we consider three groups of respondents: non-AIAN respondents from PUF, AIAN respondents identified within the PUF, and AIAN respondents included in the RO; note that all of these data are derived from the CHIS. Table 1 presents summary statistics for key outcome variables and covariates across these groups. Statistically significant differences in means between the PUF AIAN sample and the PUF non-AIAN sample (2)-(1) and between AIAN RO and the non-AIAN PUF sample (3)(1) are bolded. Shaded cells indicate a significant difference between the AIAN PUF and RO samples. We separate the analysis across these three groups as they cover distinctly different parts of the population.

##### Outcome Variables

We examine seven COVID-19-related health and economic outcomes. These outcomes capture different stages of pandemic exposure and response, ranging from infection risk to preventative health behavior and persistent health consequences.

Health-related outcomes include whether a respondent reported being fully vaccinated against COVID-19, whether they experienced symptoms lasting two or more months following infection (long-COVID), whether they had ever been tested for COVID-19, and whether they reported a positive COVID-19 test result conditional on testing.

To assess potential socioeconomic consequences of the pandemic, we also examine three indicators of economic hardship: severe food insecurity with hunger, job loss due to the pandemic, and reductions in income or work hours associated with pandemic-related disruptions. All outcome variables are measured as binary indicators based on survey responses.

In Table 1, we find that AIAN respondents (in both surveys) are more likely to have taken a test for COVID-19 and more likely to have had a positive COVID-19 test result than non-AIAN respondents. The highest rate of positivity is among the AIAN PUF at a rate of 47%. This rate is significantly different from the average rates among the non-AIAN sample in the PUF and on par with the AIAN RO sample. This table also shows that AIANs from both surveys are substantially more likely to report experiencing long-COVID symptoms than the non-AIAN population (40% vs 30%). AIAN respondents across both data sets are less likely to have received a complete set of COVID-19 vaccinations relative to the non-AIAN respondents in the PUF data; the average vaccination rates for non-AIAN were close to the average full vaccination rate for Californians at the end of 2023 which was approximately 85% (89% from CHIS).^1,29^ The data indicates that the average socio-economic background of the AIAN respondents is lower than the non-AIAN respondents; a larger proportion of the AIAN respondents (in both samples) are more likely to be below 200% of the federal poverty level (FPL). This is proxied via food access, which we include three levels of reported food security. We focus on the most extreme case of food insecurity with hunger. Finally, we show in this table that AIAN respondents in both samples are significantly more likely to report having lost their job or having had to reduce their work hours due to COVID-19, relative to non-AIAN respondents in the PUF.

##### Control Variables

We adjust for several demographic and socioeconomic characteristics that may influence COVID-19 exposure, health outcomes, and access to care. These include rural residence, gender, age, and marital status. Body weight status is measured using a binary indicator for obesity defined as body mass index (BMI) greater than or equal to 30. Obesity is included because it is associated with increased risk of severe COVID-19 outcomes and is known to vary across population groups. AIAN respondents are more likely to be obese with an obesity rate around 40% which is on par with the national average of 41% but higher than California’s average of 29% in 2022.^30^

Socioeconomic status is measured using an indicator for poverty defined as household income below 200% of the federal poverty level (FPL). Because food insecurity and employment outcomes are closely related to income, this poverty measure is included primarily in models examining COVID-19 health outcomes rather than economic hardship outcomes.

Finally, we include two indicators related to health care access and utilization: Medicare coverage and emergency department (ER) visits within the past year. These variables serve as proxies for differences in health care access and patterns of health care utilization, which may influence both the likelihood of COVID-19 testing and the detection of long-term symptoms. Prior research has documented differences in health care access between AIAN and non-AIAN populations, including greater reliance on emergency departments for care in settings with limited primary care access. We find that AIAN respondents are more likely to have visited an ER in the year prior to the survey; the rates are 21% and 24% in the PUF and RO surveys, respectively. Consistent with their younger age profile, AIANs in both samples are less likely to be covered by Medicare.

### Methodology

We estimate multivariable generalized linear models to examine associations between demographic characteristics, health status, and COVID-19-related health and economic outcomes. Because all outcome variables are binary indicators, generalized linear models with a logit link function are used to estimate adjusted associations. Separate models are estimated for each outcome of interest, including positive COVID-19 test results, reported long-COVID symptoms, COVID-19 vaccination status, COVID-19 testing behavior, severe food insecurity, job loss associated with the pandemic, and reductions in work hours or income associated with the pandemic.

All models include demographic and socioeconomic covariates that may influence both exposure to COVID-19 and access to health care. These covariates include rural residence, gender, age, marital status, obesity status, poverty status, and indicators of health care access and utilization. Indicators of health care access include Medicare coverage and emergency department visits in the previous year. Year fixed effects are included to account for differences across survey waves between 2021 and 2023.

To examine potential differences across population groups, models are estimated separately for three samples: non-AIAN respondents from the PUF, AIAN respondents identified within PUF, and AIAN respondents included in the RO. Stratified models allow the relationships between socioeconomic conditions, health status, and COVID-19 outcomes to vary across these populations, which may differ in geographic distribution, access to health services, and socioeconomic conditions.

For models examining economic hardship outcomes (severe food insecurity, job loss, and reductions in work hours or income) we additionally include COVID-19-related health outcomes as explanatory variables. These models assess whether pandemic-related health experiences, including vaccination status, positive COVID-19 test results, and long-COVID symptoms, are associated with indicators of economic hardship.

All analyses apply the survey weights provided by CHIS to account for the complex survey design and to produce population-representative estimates for California residents. Standard errors are estimated using robust variance estimators.

## Results

### Health-Related COVID-19 Outcomes

Table 2 reports adjusted associations between respondent characteristics and two COVID-19 health outcomes: reporting a positive COVID-19 test and experiencing long-COVID symptoms. Results are shown separately for non-AIAN respondents in the PUF, AIAN respondents identified in the PUF, and AIAN respondents from RO.

Across all samples (in the first three columns), rural residence is not significantly associated with the probability of reporting a positive COVID-19 test. Obesity is positively associated with reporting a positive COVID-19 test among non-AIAN respondents and shows a similar but less precisely estimated relationship among AIAN respondents. In one model, we find that poverty is associated with a lower probability of reporting a positive COVID-19 infection among respondents in the AIAN RO, while no meaningful association is observed in the PUF samples. Medicare coverage is negatively associated with reporting a positive COVID-19 test across samples.

The remaining columns of Table 2 report associations with long-COVID symptoms among respondents who reported having had COVID-19. Rural residence is positively associated with reporting long-COVID symptoms among both non-AIAN and AIAN respondents in the PUF sample. Obesity and poverty are also positively associated with reporting long-COVID symptoms, with estimated effects that are more than double for AIAN respondents than the non-AIAN respondents. Reporting being below the poverty line related to higher reported long-COVID symptoms in all three samples; the largest effect observed in the RO sample. Medicare coverage is negatively associated with reporting long-COVID symptoms, with the largest negative association observed among AIAN respondents in the restricted oversample.

### Vaccination and Testing Behavior

Table 3 presents associations between respondent characteristics and two COVID-19 preventative behaviors: full vaccination status and testing for COVID-19. Rural residence is negatively associated with the probability of reporting full COVID-19 vaccination across all samples, with the largest reductions observed among AIAN respondents. Poverty is also negatively associated with vaccination status. In contrast, Medicare coverage is positively associated with reporting full vaccination status across samples. Emergency department utilization in the prior year is not significantly associated with vaccination status among AIAN respondents but is negatively associated with vaccination among non-AIAN respondents. In the final three columns, we find that being in poverty and residing in a rural community is negatively related to having tested for COVID-19; estimated results are statistically significant for all but the rural coefficient for the AIAN PUF sample.

### Economic Outcomes

Table 4 examines associations between COVID-19-related health experiences and indicators of economic hardship, including severe food insecurity, job loss, and reductions in income or hours worked.. Panel A shows associations with severe food insecurity. Full vaccination is negatively associated with severe food insecurity, although estimates are less precisely measured among AIAN samples. Reporting long-COVID symptoms is positively associated with severe food insecurity across samples, suggesting a relationship between persistent health symptoms and economic hardship.

Panel B presents associations with job loss during the pandemic. Full vaccination is negatively associated with job loss across samples. This indicates that those who are fully vaccinated are less likely to also report having lost a job. Additionally, positive test results for COVID-19 shows a negative relationship to job loss among AIAN respondents, while no significant relationship is observed among non-AIAN respondents. Reporting long-COVID symptoms is positively associated with job loss among non-AIAN respondents.

Panel C reports associations with reductions in income or hours worked. Full vaccination is negatively associated with reductions in income or hours worked; however, only the AIAN PUF sample attains statistical significance at conventional levels. There is little evidence that testing positive for COVID-19 is associated with reductions in income or hours worked across samples. Associations between long-COVID symptoms and reductions in income or hours worked are mixed across samples.

### Robustness Analysis

To assess the robustness of our AIAN classification, we re-estimate the models, excluding respondents identified as AIAN solely through Indian Health Service coverage. Results from these models are presented in Appendix Tables A.1 and A.2. The estimated associations for the health-related outcomes and economic outcomes remain largely unchanged, indicating that the main findings are not sensitive to the inclusion of respondents identified through IHS coverage.

## Discussion

This study examines differences in COVID-19 health outcomes and economic impacts AIAN populations and non-AIAN populations in California using population-representative survey data from the CHIS. Our findings indicate that AIAN respondents reported higher rates of COVID-19 infection, long-COVID symptoms, and indicators of economic hardship relative to non-AIAN respondents. These disparities persist even after adjusting for demographic characteristics, obesity, poverty, and indicators of health care access.

Consistent with prior research documenting the disproportionate burden of COVID-19 among Indigenous populations, we find that obesity and poverty are strongly associated with both COVID-19 infection and long-COVID symptoms. These associations are particularly pronounced among AIAN respondents and among respondents living in rural areas. Lower vaccination rates among rural AIAN respondents further highlight structural barriers to preventive care that have been documented in previous studies, including geographic isolation, limited health care infrastructure, and historical mistrust of health institutions.^20^

Our results also highlight the potential economic consequences of persistent COVID-19 symptoms. Long-COVID symptoms are positively associated with indicators of economic hardship, including food insecurity and job loss, suggesting that persistent health effects may contribute to labor market disruptions. In contrast, vaccination is negatively associated with job loss and reductions in work hours or income. These patterns suggest that preventative health behaviors and persistent health symptoms may have important implications for economic stability during and after the pandemic.

The relationship between long-COVID symptoms and employment outcomes may reflect several mechanisms. Persistent symptoms such as fatigue, respiratory complications, and cognitive difficulties can reduce work capacity and limit individuals’ ability to maintain stable employment. These effects may be particularly consequential for workers in occupations requiring in-person interaction or physical labor, which are more common in some AIAN communities and may also increase exposure to infectious disease.^22^ As a result, persistent health effects associated with COVID-19 may reinforce existing economic vulnerabilities in communities that already experience higher rates of poverty and limited access to health care resources.

Our findings contribute to a growing body of literature documenting the broader social and economic consequences of the COVID-19 pandemic among Indigenous populations. While previous research has primarily focused on infection rates and mortality disparities, our analysis highlights the importance of examining persistent health symptoms and downstream economic effects. The results suggest that the long-term impacts of the pandemic may extend beyond the acute health crisis, particularly for historically marginalized populations.

Several limitations should be noted. First, the outcomes analyzed in this study rely on self-reported survey responses, which may be subject to measurement error or recall bias. Second, the cross-sectional nature of the survey data limits our ability to establish causal relationships between COVID-19 health experiences and economic outcomes. Third, the classification of AIAN respondents is constrained by available survey variables and may not capture all individuals who identify as Indigenous. However, robustness checks excluding respondents identified through Indian Health Service coverage produce similar results, suggesting that the main findings are not sensitive to alternative classification approaches.

Despite these limitations, the use of a large population-representative health survey with an oversample of AIAN communities provides rare evidence on the health and economic impacts of COVID-19 among Indigenous populations. These findings highlight the importance of improving health care access, strengthening public health infrastructure in rural and Tribal communities, and addressing structural determinants of health that continue to shape pandemic vulnerability. Policies aimed at improving vaccination access, expanding health care services, and supporting individuals experiencing long-COVID symptoms may help mitigate the long-term health and economic consequences of the pandemic for Indigenous communities.

The COVID-19 pandemic exposed and amplified longstanding health and economic inequities facing Indigenous communities in the United States. Addressing the persistent effects of long-COVID and improving access to preventative health care will be essential to reducing the long-term consequences of the pandemic among AIAN populations.

## Conclusion

This study provides new evidence on the health and economic consequences of COVID-19 among AIAN populations using population-representative survey data from California. Our findings indicate that AIAN respondents experienced higher rates of COVID-19 infection, long-COVID symptoms, and economic hardship relative to non-AIAN respondents. Obesity, poverty, and limited access to health care are strongly associated with these outcomes and contribute to persistent disparities in both health and economic vulnerability.

The results highlight the potential long-term implications of persistent COVID-19 symptoms for labor market participation and economic stability in Indigenous communities. Long-COVID is associated with indicators of economic hardship, including food insecurity and job loss, while vaccination is associated with improved employment stability. These findings suggest that pandemic-related health shocks may reinforce existing structural inequalities affecting AIAN populations, particularly in rural areas with limited health care infrastructure.

Addressing these disparities will require sustained investments in public health infrastructure, expanded access to preventive and primary health care, and policies that support economic stability for communities disproportionately affected by the pandemic. Strengthening partnerships with Tribal health systems, improving rural health care access, and expanding resources for individuals experiencing long-COVID symptoms may help mitigate the long-term health and economic consequences of COVID-19 for Indigenous communities.

This study demonstrates the importance of improving representation of Indigenous populations in population health research. Enhanced data collection efforts, including targeted survey oversamples and Indigenous data governance practices, are essential for identifying disparities and informing policies aimed at improving health outcomes among AIAN communities.

## Supplementary Material

Tables 1 to 4 are available in the Supplementary Files section.

Supplementary Files

This is a list of supplementary files associated with this preprint. Click to download.

- 1BurdenSupplement42126.docx
- Tables.docx

## Data Availability

The California Health Interview Survey public use datasets used and/or analyzed during the current study are available from the corresponding author on reasonable request. The restricted rural oversample dataset that supports the findings of this study are available from the California Health Interview Survey but restrictions apply to the availability of these data, which were used under license for the current study, and so are not publicly available. Data are however available from the authors upon reasonable request and with permission of the California Health Interview Survey.
